# Effect of curcumin, the active constituent of turmeric, on penicillin-induced epileptiform activity in rats

**Published:** 2012

**Authors:** Esmaeal Tamaddonfard, Amir Erfanparast, Nasrin Hamzeh-Gooshchi, Shahnaz Yousofizadeh

**Affiliations:** 1 *Department of Basic Sciences, Faculty of Veterinary Medicine, Urmia University, Urmia 57153-1177, I.R. Iran*

**Keywords:** Curcumin, Diazepam, Penicillin-Induced Seizures, Rats

## Abstract

**Objective: **Curcumin is a major constituent of turmeric and has many biological functions such as anticancer and anti-inflammatory effects. The present study was conducted to investigate the effects of curcumin and diazepam in separate and combined treatments on penicillin-induced seizures in rats.

**Materials and Methods: **In urethane-anesthetized rats, epileptiform activity was induced by intracortical (i.c.) administration of penicillin (200 IU, 1 µl), and frequency and amplitude of spike waves were analyzed using electrocorticographic recordings.

**Results: **Intraperitoneal (i.p.) injections of curcumin at doses of 100 and 200 mg/kg, and intracerebroventricular (i.c.v.) injection of diazepam at a dose of 5 µg significantly (p<0.05) reduced both frequency and amplitude of spike waves. Co-administrations of curcumin (50 mg/kg, i.p.) with diazepam (5 µg, i.c.v) enhanced the antiepileptic effect of diazepam (5 µg, i.c.v).

**Conclusion:** The results suggested that both curcumin and diazepam suppressed penicillin-induced epileptiform activity. A potentiation effect was observed between curcumin and diazepam in reducing penicillin-induced seizures.

## Introduction

Spices possess several medicinal properties and have been used effectively by indigenous populations in their systems of medicine (Srinivasan, 2005 ▶). Curcumin is a yellow-orange pigment extracted from turmeric, a commonly used spice, derived from the rhizome of the herb *Curcuma longa* (Maheshwari et al., 2006 ▶). It is well known that curcumin has a wide range of biological and pharmacological effects, including antioxidant, anticancer, antitumor, anti-inflammatory, antidiabetic and antimicrobial activities (Maheshwari et al., 2006 ▶; Hatcher et al., 2008 ▶). Recent studies suggest neuroprotective properties of curcumin in prevention of neurodegenerative diseases (Cole et al., 2007 ▶; Kulkarni and Dhir, 2010 ▶). Curcumin exerts its neuroprotective effects by changing the level of brain neurotransmitters and inflammatory mediators (Bishoni et al., 2008 ▶; Wang et al., 2010 ▶). Experimental evidences have shown protective effects of curcumin in various animal models of epilepsy (Bharal et al., 2008 ▶; Du et al., 2009 ▶; Gupta et al., 2009 ▶; Jyoti et al., 2009 ▶). 

Epilepsy is a complex neurological disorder characterized by recurrent seizures of cerebral origin, presenting with episodes of sensory, motor, and autonomic disturbances with or without loss of consciousness (Sridharan, 2002 ▶). Epileptic seizures result from excessive discharge in a population of hyperexcitable neurons in cortical and hippocampal structures (Avanzin and Franceschetti, 2003 ▶). Penicillin alters the excitation-inhibition balance in cortical tissues by inhibiting GABA receptors, owing to its structural resemblance to a specific GABA_A_ receptor antagonist, bicuculline, and thus leads to rhythmic epileptiform discharges (Fisher, 1989 ▶). Penicillin-induced epileptiform activity has been established as a model of seizures in rats for studying the effects of antiepileptic drugs (Tamaddonfard et al., 2012 ▶; Yildirim et al., 2011 ▶; Dragic and Pavlovic, 2004 ▶).

The present study was designed to investigate the effect of (i.p.) injections of curcumin on penicillin-induced seizures. In addition, the contribution of GABA_A_-benzodiazepine receptor system was assessed using (i.c.v.) injection of diazepam (a GABA_A_-benzodiazepine receptor agonist) with and without curcumin. 

## Materials and Methods

**Animals**

Healthy adult male Wistar rats, weighing 250-270 g were used in this study. Rats were maintained in polyethylene cages with food and water available *ad libitum* in a laboratory with controlled ambient temperature (22±0.5 °C) and under a 12 h light-dark cycle (lights on 07:00 h). Experiments were carried out between 13:00 h and 17:00 h. Six rats were used in each experiment. The experimental protocol was approved by the Veterinary Ethics Committee of the Faculty of Veterinary Medicine of Urmia University and was performed in accordance with the National Institutes of Health Guide for Care and Use of Laboratory Animals.

**Drugs**

Drugs used in the present study included urethane, curcumin, diazepam and penicillin G potassium. The drugs were purchased from Sigma–Aldrich Co., St Louis, MO, USA. Curcumin was dissolved in dimethyl sulfoxide (DMSO). A drop of Tween 80 was added to diazepam plus normal saline solution. Urethane and penicillin were dissolved in normal saline.

**Treatment groups**

The rats were divided into six groups with six rats in each group. Group A received i.p. normal saline plus (i.p.) DMSO after (i.c.) injection of penicillin. In groups B, C, and D (i.p.) injections of normal saline and curcumin at doses of 50, 100, and 200 mg/kg were performed after (i.c.) injection of penicillin, respectively. Group E was treated with (i.c.v.) injection of 5 µg of diazepam plus (i.p.) injection of DMSO after (i.c.) injection of penicillin. Group F received (i.c.v.) injection of diazepam (5 µg) followed by (i.p.) injection of curcumin (50 mg/kg) after (i.c.) injection of penicillin. Normal saline and diazepam were (i.c.v.) injected 5 min, and DMSO and curcumin were (i.p.) injected 10 min after (i.c.) injection of penicillin. The drug doses used here were selected according to the investigations in which the used doses of curcumin and diazepam were 50-200 mg/kg and 2.5-10 μg, respectively (Gupta et al., 2009 ▶; Tamaddonfard et al., 2011 ▶). 

**Study protocol**

The animals were anesthetized with (i.p.) injection of urethane (1.2 g/kg), and placed in a stereotaxic apparatus (Stoelting, Wood Lane, IL, USA). Rectal temperature was measured by a digital thermometer and was maintained between 36 and 37 °C using a controlled heating pad system. Thereafter, the scalp was incised, and the skull was leveled off around the bregma. 

The epileptic focus was produced by (i.c.) injection of penicillin (Tamaddonfard et al., 2012 ▶). Briefly, a hole with 0.8 mm in diameter was made in the right parietal bone overlying the right sensory-motor cortex (2 mm posterior to the bregma and 3 mm lateral to the midline) (Paxinos and Watson, 1997 ▶). Penicillin G potassium (200 IU, 1 μl) was injected 1.2 mm beneath the surface of the skull using a 5 μl Hamilton’s syringe in a period of 90 s (Tamaddonfard et al., 2012 ▶; Canan et al., 2008 ▶). The (i.p.) injections of DMSO and curcumin were performed in a volume of 1 ml/kg body weight using a 25-gauge injection needle. For (i.c.v.) injections of normal saline and diazepam, an additional hole with 0.8 mm in diameter was drilled in the left parietal bone according to the following coordinates: 0.8 mm posterior to the bregma and 2 mm lateral to the midline (Paxinos and Watson, 1997 ▶). The tip of the needle of a 5 μl Hamilton’s syringe was introduced through the hole into the brain and was placed at 4 mm below the surface of the skull in the left lateral ventricle of the brain. The volume of solutions to be injected into lateral ventricle was 1 µl and injection was made over a period of 30 s. The injection needle was left in place for a further 30 s after completion of the injection to facilitate diffusion of the drug. After injection, the hole was closed using acrylic cement (Acropars, Tehran, Iran). In the present study, we used (i.c.v.) injection route of administration of diazepam, because it may achieve a greater drug concentration at the epileptogenic area (Tamaddonfard et al., 2012 ▶). 

Electrocorticography (ECoG) recordings were performed as described by Tamaddonfard et al. (2012) ▶. Two 5-mm height pin electrodes (0.5 mm in diameter) were inserted into the right frontal and parietal bones according to the following coordinates: first electrode, 1 mm anterior to the bregma and 2 mm lateral to the midline (frontal electrode); second electrode, 5 mm posterior to the bregma and 2 mm lateral to the midline (parietal electrode). The common reference electrode was fixed on the left pinna. The electrodes were connected to a 4-channel physiograph (Physiograph 4-channels, MK-III-P, NARCO Bio-systems, USA) via a universal coupler (Universal coupler, type 7189, NARCO Bio-systems, USA) for ECoG activity recordings. The ECoG recordings were performed at 15 minuts before (baseline activity) and at 15, 30, 45, 60, 75, and 90 minutes after (i.c.) injection of penicillin. In each of above mentioned times, ECoG activity was recorded for a period of 1 min with two speeds (1 and 4 mm/s). To show the first spike wave, ECoG was recorded for eight min after (i.c.) injection of penicillin. The frequency and amplitude of spike waves were calculated from the recorded ECoGs. 

**Injection sites verification**

At the end of experiments, the rats were (i.c.v.) and (i.c.) injected with 10 and 1 µl methylene blue, respectively, and they were then deeply anesthetized with intracardiac high dose injection of thiopental sodium (Biochemie GmbH, Vienna, Austria) and decapitated. The brains were removed and placed in a formaldehyde (10%) solution. After 24 h, the brains were sliced into 1 mm slices and the distribution of the dye in the injection sites were controlled under a loupe. 

**Statistical analyses**

Data were analyzed by factorial analysis of variance (ANOVA) and Duncan's test. All the values are expressed as the mean±SEM. Statistical significance was set at p<0.05.

## Results

**Figure 1:**
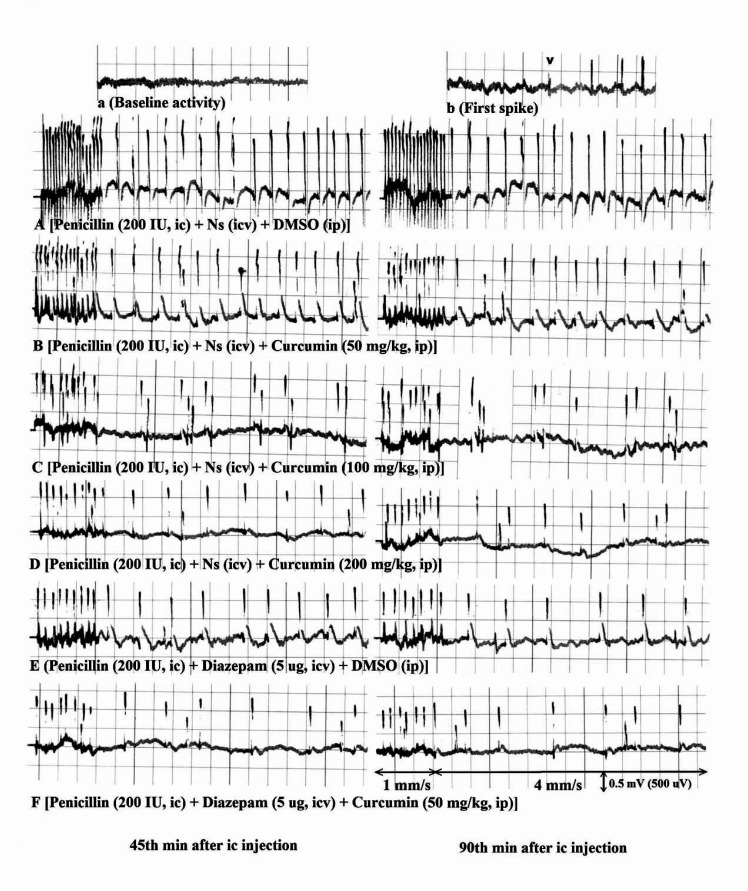
Electrocoricogram (ECoG) recording samples obtained from right sensory-motor cortex of anesthetized rats treated with curcumin and diazepam after penicillin. a) Showing the baseline activity recoded at 15 Minute before penicillin. b) Showing the first spike wave (arrow head) induced by penicillin. (**A**, **C, D, E, F)** Showing the suppressive effects of curcumin at doses of 100 and 200 mg/kg, diazepam at a dose of 5 μg and diazepam (5 μg) plus curcumin 50 mg/kg on 45 and 90 Minutes of epileptiform activity induced by penicillin, respectively. (**B**) Showing no significant effects of curcumin (50 mg/kg) on penicillin-induced epileptiform activity. Normal saline and diazepam were (i.c.v.) injected and DMSO and curcumin were (i.p.) injected 5 and 10 minutes after (i.c.) injection of penicillin. The (i.c.v.) injections of normal saline (Ns), diazepam, and (i.p.) injections of DMSO and curcumin did not change the baseline activity (data not shown). ECoG was recorded with two speeds (1 and 4 mm/s) under calibration of 0.5 mV/5 mm (i.e. 500 μV/5 mm). Ns: Normal saline, DMSO: dimethyl sulfoxide, (i.c.): intracortical, (i.c.v.): intracerebroventricular, (i.p.): intraperitoneal.

Figure 1 shows the ECoG recordings obtained from the present study. A typical recording during the basal brain activity of an anesthetized rat can be seen in Figure 1a. Although there were some individual differences between basal activity recordings among the animals, an activity dominated by slow and low amplitude wave components (20-50 μV) is a characteristic feature of such preparation. The latent or silent period began immediately after injection of penicillin and lasted for approximately 3-7 minutes. At the end of this period, first epileptiform wave abruptly appeared. The first spike wave has been shown by arrow head (Figure 1b). 

The (i.c.) injection of penicillin (200 IU) induced epileptiform activity characterized by constant levels of high frequency and high amplitude spike waves (over 300 μV) throughout the experiment (Figure 1A). Curcumin (i.p.) at a dose of 50 mg/kg had no effect (Figure 1B), but at doses of 100 and 200 mg/kg, curcumin decreased spike waves frequency and amplitude (Figures 1C and 1D). Diazepam (i.c.v) alone at a dose of 5 µg and diazepam (5 µg, i.c.v.) plus curcumin (50 mg/kg, i.p.) decreased the frequency and amplitude of spike waves (Figures 1E and 1F). 

**Figure 2:**
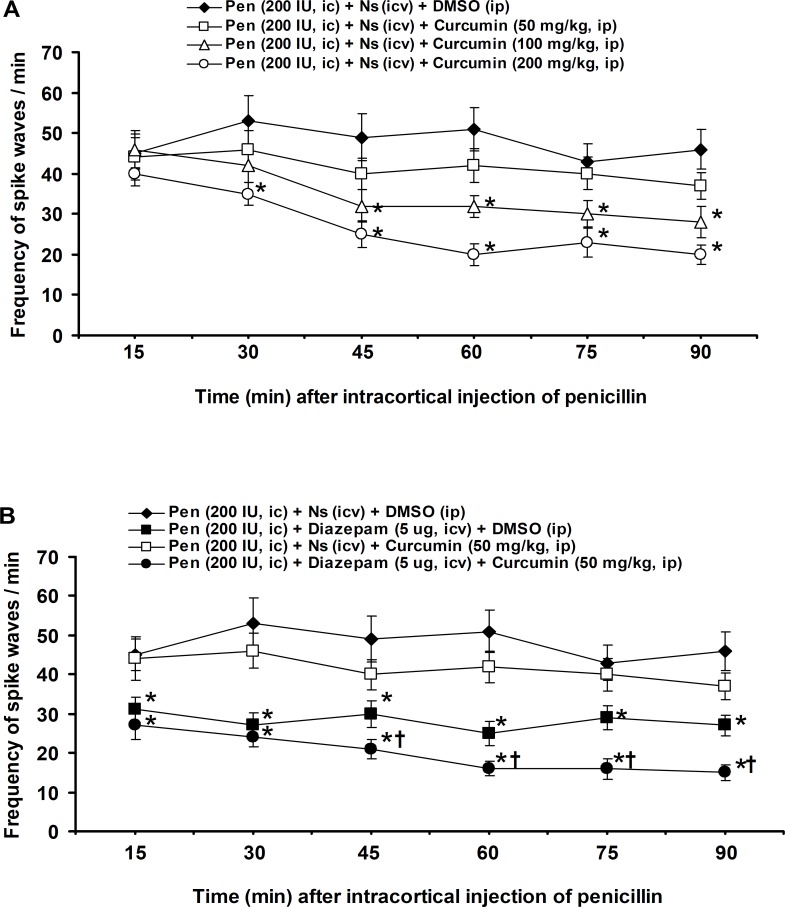
Effects of (i.p.) injections of DMSO and curcumin (**A**), (i.c.v.) injections of normal saline and diazepam and (i.c.v.) injection of diazepam plus (i.p.) injection of curcumin (**B**) on the frequency of spike waves induced by (i.c.) injection of penicillin in rats. All values are expressed as the mean±SEM (n=6). Statistical comparisons among groups were carried by factorial ANOVA followed by Duncan's test. *p<0.05 in comparison with normal saline, ^†^p<0.05 in comparison with diazepam (5 μg). Pen: Penicillin, Ns: Normal saline, DMSO: dimethyl sulfoxide, (i.c.): intracortical, (i.c.v.): intracerebroventricular, (i.p.): intraperitoneal.

The frequency of spike waves was 42±4 at 15 minute after (i.c.) application of penicillin and with a constant level reached to 46±4.9 at the end of experiment (90 minute) (Figures 2A and 2B). The (i.p.) injection of curcumin at a dose of 50 mg/kg produced no significant effect, but at doses of 100 and 200 mg/kg, curcumin significantly (p<0.05) decreased the frequency of spike waves at 45-90 and 30–90 minutes after (i.c.) injection of penicillin, respectively (Figure 2A). The (i.c.v.) injection of diazepam at a dose of 5 µg significantly (p<0.05) reduced 15–90 minutes of the frequency of spike waves (Figure 2B). The (i.p.) injection of curcumin (50 mg/kg) after (i.c.v.) injection of diazepam (5 µg) significantly (p<0.05) enhanced the 45-90 minutes suppression of frequency of spike waves induced by diazepam (5 µg) (Figure 2B). 

**Figure 3:**
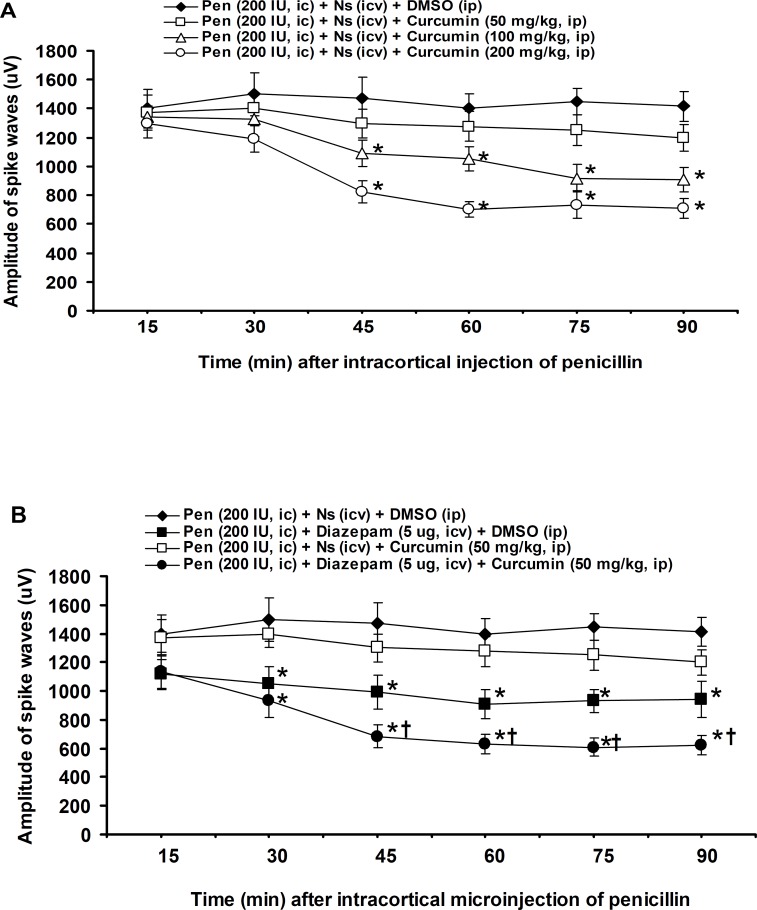
Effects of (i.p.) injections of DMSO and curcumin (**A**), (i.c.v.) injections of normal saline and diazepam and (i.c.v.) injection of diazepam plus (i.p.) injection of curcumin (**B**) on the amplitude of spike waves induced by (i.c.) injection of penicillin in rats. All values are expressed as the mean±SEM (n=6). Statistical comparisons among groups were carried by factorial ANOVA followed by Duncan's test. *p<0.05 in comparison with normal saline, ^†^p<0.05 in comparison with diazepam (5 μg). Pen: Penicillin, Ns: Normal saline, DMSO: dimethyl sulfoxide, (i.c.): intracortical, (i.c.v.): intracerebroventricular, (i.p.): intraperitoneal

The amplitude of spike waves was 1400±134 μV at 15 minute after (i.c.) injection of penicillin and with a constant level reached to 1416±102 μV at the end of experiment (90 minute) (Figures 3A and 3B). The (i.p.) injection of curcumin at a dose of 50 mg/kg produced no significant effect, but 45-90 minutes of the amplitude of spike waves were significantly (p<0.05) decreased by 100 and 200 mg/kg of curcumin (Figure 3A). The (i.c.v.) injection of diazepam at a dose of 5 µg significantly (p<0.05) reduced 30-90 minutes of the amplitude of spike waves (Figure 3B). The (i.p.) injection of curcumin (50 mg/kg) after (i.c.v.) injection of diazepam (5 µg) significantly (p<0.05) enhanced the 45-90 minutes suppression of amplitude of spike waves induced by diazepam (5 µg) (Figure 3B).

## Discussion

The present results showed that (i.c.) injection of penicillin (200 IU, 1μl) produced an epileptiform ECoG activity characterized by high frequency and amplitude spike waves. Our results (frequency and amplitude of spike waves after (i.c.) injection of 200 IU of penicillin) are approximately consistent with other findings in which the doses of (i.c.) injected penicillin were 200-500 IU (Canan et al., 2008 ▶; Cakil et al., 2011 ▶; Tamaddonfard et al., 2012 ▶). Clinical experience has indicated that high systemic doses of penicillin in humans can produce myoclonus, generalized tonic-clonic seizures and encephalopathy (Fossieck and Parker, 1974 ▶). In addition, it is well known that (i.c.) or systemically administered penicillin results in a prominent epileptiform activity both electrophysiologically and behaviorally in laboratory animals (Chen et al., 1986 ▶; Fisher, 1989 ▶). 

In the present study, curcumin (100 and 200 mg/kg, i.p.) and diazepam (5 µg, i.c.v.) produced antiepileptic effects. Moreover, a sub-therapeutic dose of curcumin (50 mg/kg, i.p.) enhanced the antiepileptic effect of diazepam (5 µg, i.c.v.). These results indicate that curcumin and diazepam may produce the same antiepileptic effects on penicillin-induced epilepsy. In addition, a potentiation effect was observed between curcumin and diazepam in producing an antiepileptic effect against penicillin. Curcumin has been exerted antiepileptic effects in various models of experimentally-induced epilepsy. Curcumin (300 mg/kg, i.p.) suppressed the death of hippocampal neurons induced by generalized seizures in pilocarpine-kindled rats (Huang et al., 2006 ▶). Dietary intake of curcumin inhibited the onset and progression of seizures induced by intracortical microinjection of FeCl_3_ in rats (Jyoti et al., 2009 ▶). The (i.p.) injection of curcumin at doses of 100 and 200 mg/kg reduced the percent incidence of convulsions as well as the increased brain malondialdehyde levels induced by kainic acid, an analogue of glutamic acid, in rats (Gupta et al., 2009 ▶). The low molecular weight and polar structure of curcumin allows it to penetrate the blood-brain barrier effectively after oral and systemic use (Choi et al., 2011 ▶; Suresh and Srinivasan, 2010 ▶). Diazepam and other benzodiazepines mostly exert their pharmacological actions by allosterically modulating the GABA_A_-benzodiazepine receptor complex to produce a facilitation effect on the GABA-mediated inhibitory neurotransmission in the central nervous system (Macdonald and Olsen, 1994). Intravenous (i.v.) and (i.c.v.) injections of diazepam reduced frequency and amplitude of spike waves induced by (i.c.) injection of penicillin in rats (Gartside, 1978 ▶; Tamaddonfard et al., 2012 ▶). 

There are some interactions between curcumin and diazepam. Using elevated plus maze for evaluation of anxiety, it has been revealed that long-term (14 days) oral administration of curcumin (10 mg/kg) and diazepam (3 mg/kg) produced the same anxiolytic effects on pentylentetrazole-induced anxiety-like behavior (Chimakurthy and Talasila, 2010 ▶). The opposite effects were observed between curcumin and diazepam when the passive avoidance retention test for memory evaluation was used (Chimakurthy and Talasila, 2010 ▶). Abid et al. (2011) ▶ reported that the hydroalcoholic extract of polyherbal formulation (HAEPHF) which consists of *Curcma longa* and *Butea frondosa* produced the same sedative, antianxiety, and muscle relaxant effects as diazepam did. The involvement of the GABA_A_-benzodiazepine receptor system has been reported in the antiepileptic effects of the ethanolic leaf extracts of *Rhus tridentate*, *Rhus rehmanniana*, and *Hoslundia opposite* and the ethanolic corm extract of *Hypoxis clochicifolia* (Risa et al., 2004 ▶). Although the levels of oxidative stress markers were not measured in the present study, the antiepileptic activity of a combination of diazepam and curcumin observed in the present study might be associated with their antioxidant activities. The antioxidant effect of curcumin is well established (Maheshwari et al., 2006 ▶; Hatcher et al., 2008 ▶). It has been reported that a single dose of diazepam injected (i.p.) reduced the levels of lipid peroxidation and produced a mild preservation effect on superoxide dismutase activity in the striatum of the brain (Méndez-Cuesta et al., 2011). The involvement of reactive oxygen species in epileptic seizures is well known ().

In conclusion, the results of the present study indicate that both curcumin and diazepam produced the same antiepileptic effects in penicillin-induced epilepsy. Moreover, a potentiation effect was observed between curcumin and diazepam in producing antiepileptic effect. 
